# A Novel Driving Noise Analysis Method for On-Road Traffic Detection

**DOI:** 10.3390/s22114230

**Published:** 2022-06-01

**Authors:** Qinglu Ma, Lian Ma, Fengjie Liu, Daniel (Jian) Sun

**Affiliations:** 1 School of Traffic and Transportation, Chongqing Jiaotong University, Chongqing 400074, China; mql@cqu.edu.cn (Q.M.); lfj@mails.cqjtu.edu.cn (F.L.); 2 School of Future Transportation, University of Chang’an, Xi’an 710064, China; danielsun@sjtu.edu.cn

**Keywords:** traffic detection, traffic noise, feature extraction, Triangular Wave Analysis

## Abstract

Effective noise reduction and abnormal feature extraction are important for abnormal sound detection occurring in urban traffic operations. However, to improve the detection accuracy of continuous traffic flow and even overlapping vehicle bodies, effective methods capable to achieve accurate signal-to-noise ratio and appropriate characteristic parameters should be explored. In view of the disadvantages of traditional traffic detection methods, such as Short-Time Energy (STE) and Mel Frequency Cepstral Coefficients (MFCC), this study adopts an improved spectral subtraction method to analyze traffic noise. Through the feature fusion of STE and MFCC coefficients, an innovative feature parameter, E-MFCC, is obtained, assisting to propose a traffic noise detection solution based on Triangular Wave Analysis (TWA). APP Designer in MATLAB was used to establish a traffic detection simulation platform. The experimental results showed that compared with the accuracies of traffic detection using the traditional STE and MFCC methods as 67.77% and 76.01%, respectively, the detection accuracy of the proposed TWA is significantly improved, attaining 91%. The results demonstrated the effectiveness of the traffic detection method proposed in solving the overlapping problem, thus achieving accurate detection of road traffic volume and improving the efficiency of road operation.

## 1. Introduction

In many cities throughout of the world, the existing road network infrastructure needs to be maintained and improved, such as widening existing roads and making use of intelligent transportation system technologies. The number of on-road sensors were usually huge, and could not capture all traffic flow conditions for short-term traffic flow predictions. At the same time, traffic noise emissions may be related to the following attributes: e.g., power unit component; the interaction between speed, traffic flow type and road slope; rolling noise component; and the relation function between traffic speed and road surface [1]. As one of the major input data sources, a traffic noise spectral profile was used for the real-time traffic detection to obtain road traffic flow estimation in real time, with an average correct classification rate of about 96% [2]. Taking audio signals from two nearby sensors, the generalized cross correlation (GCC) function was combined with a particle filter to jointly estimate speed and wheelbase length. Using voice for road traffic monitoring, a design method for the microphone array was put forward based on the correlation function of acoustic observation vehicle trajectory [3]. Probabilistic noise models with more explicit road surface maker (RSM) features were developed to analyze the results of the RSM feature detection under various driving conditions [4]. Based on the probabilistic sensor model, an RSM model designed by Jo et al. a utilized particle filter to update the measurements, thereby improving the localization performance [5]. By extracting the peak power envelope in the traffic noise signal, Torija used a microphone array to record the sound of vehicles and estimate the number of vehicles, in which lanes were divided according to the signal propagation time [6]. Audio sensors were used for traffic volume collection based on existing traffic detection sensors (coil and video detectors), and Support Vector Regression was introduced for audio data process [7]. Based on the traditional traffic noise prediction models and the improvement, it was stipulated that an efficient strategy processed a dual-channel audio record with a portable system. Analyzing the mechanism of the vehicle signal collected in detail, Steele et al. proposed a blind separation method to improve the classification of traffic noise [8,9]. To this end, a stereo signal recording system was proposed for detecting vehicles passing by and vehicle types (motorcycle, ordinary car and large truck) [10]. In order to develop an artificial neural network (ANN) model for predicting highway traffic noise, a method using the Levenberg-Marquardt (L-M) algorithm was proposed to train the multi-layer feed-forward back-propagation (BP) neural network. The results show that the ANN, a powerful traffic noise modeling technique, has a much smaller percentage difference than regression analysis [11]. Aiming at combining the genetic algorithm with machine learning, focusing on the features of traffic noise selection and vehicle classification, the correct classification rate was finally largely improved [12]. Based on MFCC representation to classify traffic flows of three different density levels, Borkar et al. developed a neuro-fuzzy classifier that achieved a classification of over 95% [13]. Linear SVM was used to extract vehicles from all approaching vehicles detected by radar sensors; meanwhile, hierarchical clustering was used to classify different traffic patterns according to the time series [14]. Emphasizing that traffic detection based on traffic sound is gradually becoming an important issue in the field of traffic flow parameter acquisition [15], when utilizing the noise differences perceived by passing road vehicles (approach, pass and departure), the major challenge is to improve the recognition accuracy of road noise events. By preprocessing the vehicle signal and extracting the features, Nooralahiyan et al. used the directional microphone to collect four vehicles: a small saloon (1300 VW), a medium saloon (2L Ford), a 250 cc motorcycle, and a Ford Diesel. Without controlling vehicle speed or background noise, the same equipment and 700 roadside recordings were made at road sites in the city, in which parameter transformation based on autocorrelation analysis was used to preprocess vehicle signals and extract features [16]. On the grounds of acoustics principle, Schclar et al. proposed a real-time vehicle automatic detection algorithm, which extracted spatiotemporal features from records using wavelet packet transform, and these features constituted the unique acoustic features of each recording [17]. Traffic noise signals were used to measure vehicle speed, and then the cruise speed is detected according to the magnitude relationship between the vehicle speed and the Doppler frequency shift. The traffic flow volume and vehicle types on different road levels were obtained to determine the weight of various noise influencing factors [18,19]. Due to the significant impact of driving signal feature selection on the performance of vehicle detection and identification, some essential features that can effectively represent vehicle signals need be extracted. Dinesh and Naveen introduce two different types of characteristics of traffic noise, Meir Frequency Cepstrum Coefficient and Wavelet Packet Transform, respectively. For binary traffic scene classification, MFCC features are up to 100% [20]. To solve the problem of poor recognition rate of overlapping noise signals, Dennis et al. extracted the local spectral features of noise signals, thus separating background noise and identify overlapping acoustic events, so as to provide a reference approach for overlapping signal recognition studies [21]. In the traffic detection method based on spectrum analysis, vehicle noise detection signals along certain corridor passages was obtained to perform spectrum detection and feature separation of multiple endpoints at the same time, thereby realizing the separation of overlapping vehicle bodies in congested road sections [22]. A smart phone-based sound measurement calibration protocol was tested under real conditions using a group of eight smart phones. Comparison with class 1 sound level meters at six control points showed an average error of −0.6 ± 1.2 dB for all handsets [23]. In the test area, 24 monitoring sensors were installed and six dynamic noise maps were obtained. By associating the prediction error of traffic noise with the error generated by the traffic flow model, Dynamap prediction was successfully improved, and the overall error was limited within about 3 dB [24]. Using the local spectral features of sound to identify overlapping sound events and separate the background noise has a certain effect on the recognition of overlapping signals, and can be applied to the situation of overlapping traffic noise. 

This paper proposed an improved spectral subtraction method to deal with traffic noise. A new feature parameter E-MFCC was defined through the feature fusion of STE and MFCC coefficient, so that a new traffic detection method based on traffic noise is proposed in the frame of TWA, to improve traffic detection accuracy. The remainder of the paper is structured as follows: Section 2.1 introduces the principle of traffic detection based on traffic noise, including pretreatments and analyses on the collected traffic noise data. Section 2.2 analyzes the characteristic parameters. With the field collected experimental data, the detection performance of the three traffic detection methods was assessed based on the experimental results in Section 2.3, so as to verify the effectiveness of the detection method. Finally, conclusions and future research directions are provided in Section 3.

## 2. Materials and Methods

### 2.1. Pretreatment and Characteristic Analysis of Driving Noise

**Figure 1:**
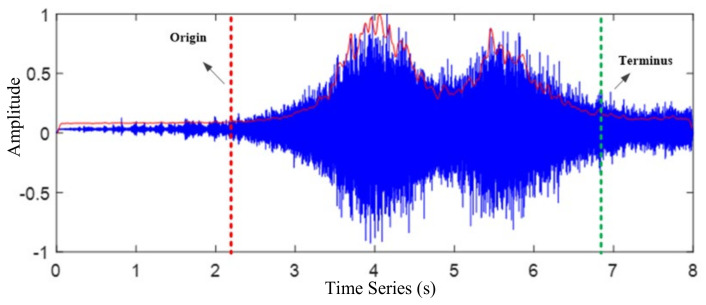
Traffic noise signal analysis.

Voice endpoint detection (VED) technology has been widely used in voice detection [25], with the main purpose being to distinguish voice and non-voice segments from the input signal. The core of using endpoint detection technology to detect traffic volume is to set thresholds and confirm vehicle signal frames. The existence of a vehicle is determined by the threshold value, as shown in Figure 1. According to the corresponding abscissa of the waveform diagram, the oversaturated road segment can be roughly determined. The red dot line within the figure represents the starting point of the vehicle road, and the green line is the ending point, both of which are roughly judged by the waveform of the driving noise signal. In this study, the low signal-to-noise ratio is not recommended to be judged out according to the waveform. For example, two vehicles were included in Figure 1, but unfortunately, only one was detected at this time. Therefore, it is necessary to set up a threshold, as shown in Figure 1, by which two sections of the waveform may be obtained.

#### 2.1.1. Pretreatment

Borkar et al. extracted the driving noise MFCC, and based on the Neuro-Fuzzy Classifier characterized by MFCC, SVM was used to classify of low traffic (40 km/h), medium traffic (20–40 km/h) and high traffic (0–20 km/h) at three different traffic density levels, showing that the classification accuracy was over 95% [26]. Kaur et al. collected traffic noise data in a ‘busy street’ and a ‘quiet street’, respectively, and extracted various time- and frequency-based features such as short-term zero crossing rate (ZCR), short-term energy (STE), root mean Square (RMS) and MFCC, yielding results with a better classification accuracy of 91.8% with Neural Network and 93% with SVM [27]. 

**Figure 2:**
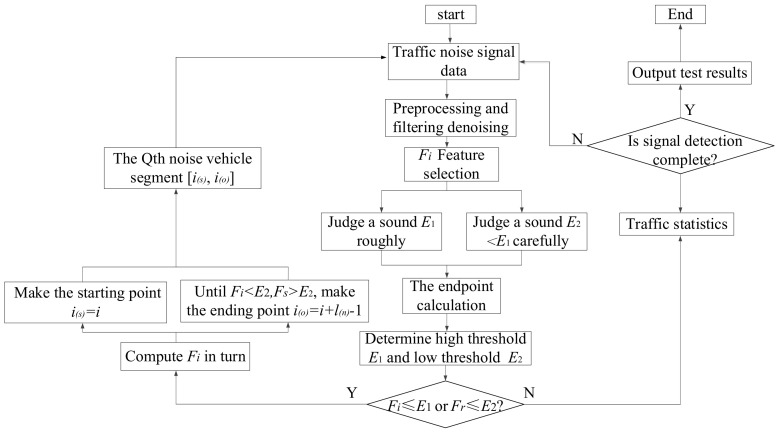
Flow chart of traffic detection and analysis algorithm.

Vehicles driving on the road are often accompanied by the influence of abnormal short-term signals, such as vehicle whistle, emergency brake, etc., which may also be erroneously detected as vehicles passing. According to the high and low thresholds, the vehicle noise signal detection and analysis algorithm was proposed in Figure 2.

Six steps are included in the algorithm as follows:

**Step 1**: The original traffic noise signal collected in real time on the road is imported into the processing system; 

**Step 2**: The collected traffic noise signal is preprocessed and filtered to obtain smooth traffic noise signal data; 

**Step 3**: Feature vectors are extracted as the configuration parameters and the eigenvalue *F_i_* of each frame signal of driving noise is calculated successively; 

**Step 4**: The high threshold is calculated as $E_{1}=(a+0.2)*\underset{1\leq j\leq n}{\max E_{j}}$, the low threshold is calculated as $E_{2}=a*\underset{1\leq j\leq n}{\max E_{j}}$ 0≤a≤10, and increased by 0.1 per time so as to set up the minimum car signal frame; 

**Step 5**: If *F_i_*≤*E*_1_ and *F_r_* ≤ *E*_2_, (*r* = *i* + 1, *i* + 2, ⋯, *i* + *l*_(*v*)_ *−* 1), then *i*_(*s*)_ = *i*, where *l*_(*v*)_ is the minimum length of the vehicle segment and *i*_(*s*)_ is the initial endpoint of the Q^th^ driving noise segment; then, *F_i_* is successively calculated from *i* = *i* + *l* until *F_i_* < *E*_2_ and *F_s_* < *E*_2_, (*s* = *i +* 1, *i +* 2, ⋯, *i* + *l*_(*n*)_ *−* 1); then, *i*_(*o*)_ = *i* + *l*_(*n*)_ *−* 1, where *l*_(*n*)_ is the minimum length of the environmental noise segment, and *i*_(*s*)_ is the end point of the *Q^th^* driving noise segment; and the interval [*i_s_*, *i_o_*] represents the *Q^th^* driving noise segment. Repeat the preceding steps; 

**Step 6**: The entire algorithm iterates between the detection state and vehicle signal state. To complete a cycle is to detect a vehicle passing, and the number of cycles equals the number of vehicles. By exploring all data sequences, the objective of road traffic flow detection can be achieved. 

The preprocessing process mainly uses the following operation modes: pre-emphasis, windowing, framing, normalization and noise reduction [27]. After sampling the noise signal, a FIR high-pass filter called pre-emphasis of audio samples, is inserted to facilitate the analysis of audio samples. The purpose is to increase the high frequency resolution of the audio signal. Pre-emphasis processing has a certain inhibitory effect on the low frequency signal. After pre-emphasis, the high frequency component of the traffic noise signal is significantly increased, and the overall amplitude of the signal becomes smaller. The waveform of the traffic noise signal after pre-emphasis becomes smoother when there are no cars, which is beneficial to the subsequent signal processing and feature extraction. 

#### 2.1.2. Framing and Windowing

The traffic noise signal is a random signal with non-stationarity, which can be regarded as a quasi-steady state process within a rather short time range. During the traffic noise signal processing, the entire signal processing needs to be framed, generally in the range of 10–30 ms, to ensure the stability of the input signal. To this end, a traffic noise signal with length *L* is framed according to Equation (1).

(1) $$\left\{\begin{array}{l} f=\sum_{i=1}^{n}\frac{L-X_{i}}{T_{i}}\\ N_{i}=X_{i}-T_{i} \end{array}\right.$$

where f is the total number of frames after splitting, *L* is the signal length, $T_{i}$ is the displacement of i+1^th^ frame to i^th^ frame, namely the frame shift, $X_{i}$ is the overlapping part between the two frames, $N_{i}$ is the frame length and $X_{i}=N_{i}-T_{i}$, and the final data is divided into *f* frames.

#### 2.1.3. Normalized Processing

During driving noise signal collection, even if the same vehicle is affected by factors such as speed and location of acquisition equipment, the signal amplitude collected is different. To eliminate the influence of various factors on traffic detection, when the position of the weak traffic signal in the strong signal is obtained, a selection judgment is required. Euclidean distance is used as the judgment function. Because of the output of continuous signals in the audio, the collected signal amplitude is used to eliminate the influence and weight of the location of the collection device by using the distance discrimination index, and the expression is shown in Equation (2):

(2) $$\begin{array}{l} D(i,j)=\sum_{m=1}^{M}{\sum_{n=1}^{N}\left[X^{ij}(m,n)\right]}^{2}-2\sum_{m=1}^{M}\sum_{n=1}^{N}X^{ij}(m,n)Y(m,n)\\ \;\;\;+{\sum_{m=1}^{M}\sum_{n=1}^{N}\left[Y(m,n)\right]}^{2} \end{array}$$

$X^{ij}(m,n)$, Y(m,n) is the driving signal at any point in the selected area. After selecting the template, $\sum_{m=1}^{M}{\sum_{n=1}^{N}\left[X(m,n)\right]}^{2}$, ${\sum_{m=1}^{M}\sum_{n=1}^{N}\left[Y(m,n)\right]}^{2}$ is fixed; if D=0, the signal is equal; If D > 0, the normalization is shown in Equation (3):

(3) $$R(i,j)=\frac{\sum_{m=1}^{M}\sum_{n=1}^{N}X^{ij}(m,n)Y(m,n)}{\sqrt{\sum_{m=1}^{M}\sum_{n=1}^{N}\left[X^{ij}(m,n)\right]}\sqrt{\sum_{m=1}^{M}{\sum_{n=1}^{N}\left[Y(m,n)\right]}^{2}}}$$

The normalization process eliminates the number level difference between the data of each dimension and avoids large calculation errors caused by the large difference of the data level of the feature data. To analyze the collected road vehicle data accurately, noise reduction is needed. 

#### 2.1.4. Improved Spectral Subtraction Noise Reduction

**Figure 3:**
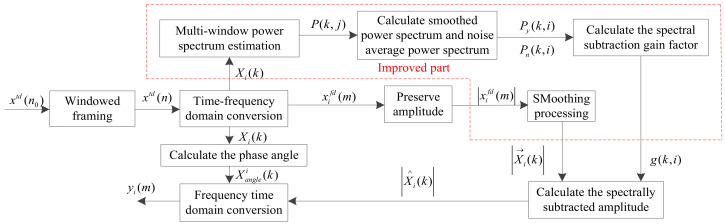
Improved spectral subtraction.

In the process of spectral subtraction, it is necessary to determine the length of the leading noise segment and the value of the sum of the parameters. The acquisition of an audio signal is completely random background noise; thus, when using spectral subtraction, it is likely the line value will be greater than this one. In this case, if spectral subtraction is used to reduce noise, the background noise environment cannot be removed and many burr peak points will be retained, which greatly reduces the effect of noise reduction. The flow chart of this method is shown in Figure 3. The improved spectral subtraction of multi-window spectral estimation is mainly based on the basic spectral subtraction technology. An orthogonal data window is improved to multiple orthogonal data windows, and the red dot line within Figure 3 represents the improved part.

The original traffic noise signal time domain sequence is *x^td^*(*n_0_*), the signal sequence after pretreatment is *x^td^*(*n*) and *x^td^*(*n*) is expressed as the i*^th^* frame traffic noise signal. The general spectral subtraction noise reduction steps include time-frequency domain conversion, noise estimation, phase angle calculation, spectral subtraction and the five steps of frequency-time domain conversion. The three steps of time-frequency domain conversion, noise estimation and phase angle calculation are important to the pre-work of spectral subtraction, as shown in Equation (4) below:

(4) xifdm=∑n=0N−1xitd(n)exp(−j2πnmN)D(m)=1NIS∑i=1NISxifdm2xifd(angle)m=arctanlm(xifdm)Re(xifdm)

where $x_{i}^{fd}\left(m\right)$ is the frequency domain signal of $x_{i}^{td}\left(n\right)$ after fast Fourier transform, *m* = 0, 1, …, *N* – 1, *D*(*m*) is the average energy of the leading non-vehicle signal segment in the collected traffic noise signal, *IS* is the time length of the leading noise segment (composed of the leading non-vehicle segment) and *NIS* is the corresponding frame number. The noise signal amplitude is $\left|x_{i}^{fd}\left(m\right)\right|$. $x_{i}^{f_{1}}\left(m\right)$ represents the phase angle of frame *i* of $x_{i}^{fd}\left(m\right)$, $\mathrm{lm}(x_{i}^{fd}\left(m\right))$ is the imaginary part of $x_{i}^{fd}\left(m\right)$ and Re(xifdm) is the real part.

The spectral subtraction process reduces the energy of the frequency domain signal $x_{i}^{fd}\left(m\right)$ and the average noise energy of each frame, and the amplitude value $\left|x_{i}^{ss}\left(m\right)\right|$ after spectral subtraction is realized as follows in Equation (5):

(5) $${\left|x_{i}^{ss}\left(m\right)\right|}^{2}=\left\{\begin{array}{ll} {\left|x_{i}^{fd}\left(m\right)\right|}^{2}-a{\left|D(m)\right|}^{2} & ,\;{\left|x_{i}^{fd}\left(m\right)\right|}^{2}-{\left|D(m)\right|}^{2}>0\\ b{\left|D(m)\right|}^{2} & ,\;\mathrm{else} \end{array}\right.$$

where *a* is a minus factor and *b* is a gain compensation factor. Then, the signal sequence *y_i_*(*k*) after spectral subtraction is obtained by combining them. The basic spectral subtraction only uses one data window in the process of noise reduction, and it is improved through multi-window spectral estimation. The flow chart of this method is shown in Figure 3.

(6) $$\left\{\begin{array}{l} P(k,i)=\mathrm{PMTM}\left[x^{td}(n)\right]\\ P_{y}(k,i)=\frac{1}{2M+1}\sum_{j=-M}^{M}P(k,i+j)\\ |{\vec{X}}_{i}(k)|=\frac{1}{2M+1}\sum_{j=-M}^{M}\left|X_{i+j}(k)\right| \end{array}\right.$$

where the multi-window spectral power spectral density P(k,i) and $P_{y}(k,i)$ are the multi-window spectral power density and the smoothed power spectral density of the kth spectral line of the *i*^th^ frame. PMTM means to estimate the multi-window spectral power spectral density and the first and last M frames of the *i^th^* frame as the center. Then, 2*M* + 1 frames are averaged. $\left|{\vec{X}}_{i}(k)\right|$ is the average magnitude spectrum, g(k,i) is the gain factor and the magnitude spectrum after spectral subtraction is $\left|\overset{\wedge }{X_{i}}(k)\right|$.

**Figure 4:**
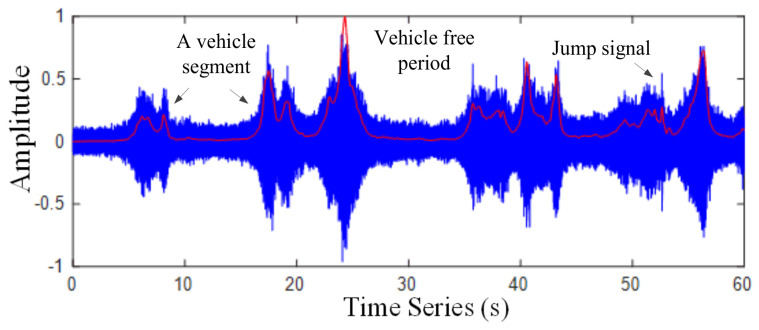
Waveform of traffic noise signal and its STE.

The short time energy of the traffic noise signal $y_{i}(n)$ in *i^th^* frame is calculated as shown in Equation (7):

(7) $$E(i)=\sum_{n=0}^{L-1}y_{i}^{2}(n)$$

where $y_{i}(n)$ is the value of a frame and n=1,2,…,L,  1≤i≤fn. L is the frame length and the square sum of the amplitude of the i*^th^* frame signal is the STE value of the corresponding time point of the signal. Figure 4 represents the original waveform of a section of traffic noise and the corresponding STE. In Figure 4, the STE of the traffic noise signal changes with time, and the energy difference between the congested segments with and without car is significant.

**Figure 5:**
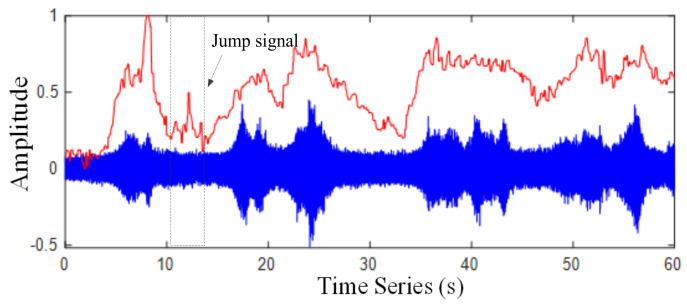
Waveform of traffic noise signal and the corresponding zero crossing rate.

In the case of great environmental noise, the existence of congested segments should be identified, so that the STE feature extraction algorithm becomes relatively easy to implement, which saves the processing time in the case of large amounts of data. Zero Crossing Rate (ZCR) indicates the number of times a voice signal waveform passes through the horizontal axis (zero level) in a frame of speech [28]. The definition of short-time average zero crossing rate is shown in Equation (8):

(8) $$Z(i)=\frac{1}{2N}\sum_{n=0}^{L-1}\left|\mathrm{sgn}[y_{i}(n)]-\mathrm{sgn}[y_{i}(n-1)]\right|$$

where sgn[⋅] is a sign function and sgn[x] = 1, when x≥0; otherwise, sgn[x] = −1. N is the length of the selected window. The original waveform of a section of traffic noise and the corresponding Zero Crossing Rate are shown in Figure 5.

The sonogram reflects the dynamic spectrum characteristics of the sound signal, which plays an important role in signal processing [29]. In the sonogram, the abscissa represents the time and the ordinate represents the amplitude. Because the three-dimensional information is mapped to the two-dimensional plane, the amplitude is expressed by the depth of the color. The deeper color indicates large amplitude at this time. The result of FFT of traffic noise signal is shown in Equation (9) below:

(9) $$Y_{i}\left(k\right)=\sum_{n=1}^{N}y_{i}\left(n-1\right)\exp\left(-j\frac{2\pi (n-1)(k-1)}{N}\right)$$

**Figure 6:**
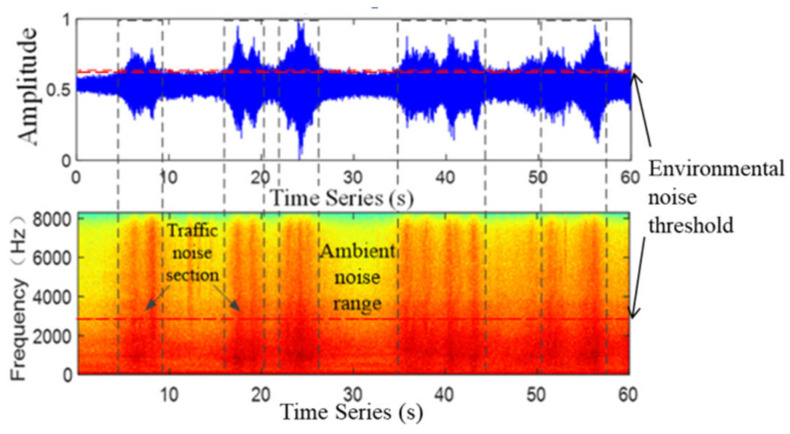
Waveform of traffic noise signal and its sonogram.

The spectrum characteristics of traffic noise signal calculated can be expressed by the matrix shown in Equation (10).

(10) $$A_{sv}={\left[a_{i}\right]}_{m\times L}=\left[\begin{array}{cccc} \left|Y_{1}(1)\right| & \left|Y_{2}(2)\right| & \ldots & \left|Y_{L}(1)\right|\\ \left|Y_{1}(2)\right| & \left|Y_{2}(2)\right| & \ldots & \left|Y_{L}(2)\right|\\ \ldots & \ldots & \ldots & \ldots \\ \left|Y_{1}(m)\right| & \left|Y_{2}(m)\right| & \ldots & \left|Y_{L}(m)\right| \end{array}\right]$$

where $A_{sv}$ represents the spectrum feature and $a_{i}$ represents the amplitude value of the traffic noise signal after FFT transformation. $m=1,2,\ldots ,{\left[N/2\right]}_{\mathrm{int}}+1$, and when the *k* indicates 1, $\left|Y_{i}(k)\right|$ and $\left|Y_{i}(N-k+2)\right|$ is equal, only $\left({\left[N/2\right]}_{\mathrm{int}}+1\right)$ sample points are needed. According to Equations (8) and (9), the spectrum feature of the traffic noise signal is extracted, as shown in Figure 6.

The Figure 6 represents a section of traffic noise signal waveform and its corresponding spectrogram extraction results, respectively. The frequency of the signal collected in the road environment is found below 8000 Hz, and the color of the traffic noise section is deeper than that of the ambient noise section. The traffic noise information is continuously distributed from the low frequency region to the medium and high frequency region, whereas the environmental noise information is mainly concentrated in the low and medium frequency region. It can be figured out from Figure 6 that above the intermediate frequency region of about 3000 Hz, the frequency distribution of the environmental noise signal is rather small, and the frequency relative to the traffic noise signal can be ignored. Therefore, the existence of congested segments can be easily judged by setting the frequency threshold.

### 2.2. Traffic Detection Based on Triangle Wave Analysis of Traffic Noise

#### 2.2.1. Feature Extraction and Fusion

This study proposes the Triangular Wave Analysis (TWA) technology for traffic volume processing. Through the analyses of STE and MFCC characteristic traffic detection methods in [30], both methods have certain limitations in detecting the performance of overlapping congested segments. The indicator of STE (*E_i_*) of the traffic noise signal is calculated by taking $d_{m}^{0}(i,n)$ from $d_{m}(i,n)$ in MFCC, and the first and last two frames of the short-time energy *E_i_* of the traffic noise signal are discarded because the frames were not included in calculating $d_{m}(i,n)$. To match the length of $d_{m}^{0}(i,n)$, the short-time energy *E_i_* is also discarded. The *E_i_* is then multiplied by the index, that is, the characteristics of the frame i signal of *E-MFCC*. 

E-MFCC does not need to set the front NIS frame audio by the MFCC describing the background noise, so as to calculate the average. The E-MFCC feature is feasible for traffic detection, which is superior to Short Term Energy and MFCC.

#### 2.2.2. Extremum Extraction

For the extracted new feature envelope, the extremum extraction method searches the local extremum points of the numerical sequence to extract the extremum of the original sequence. The new feature E-MFCC is taken as the original data of the traffic noise signal after digitization. Assuming that the original data matrix is X, Equation (11) can be expressed as follows:

(11) $$X={\left[\begin{array}{l} T_{1},T_{2},\cdot \cdot \cdot ,T_{i},\cdot \cdot \cdot ,T_{m}\\ P_{1},P_{2},\cdot \cdot \cdot ,P_{i},\cdot \cdot \cdot ,P_{m} \end{array}\right]}^{T}$$

where X is the original data matrix, i=1,2,⋅⋅⋅,m, $T_{i}$, $P_{i}$ are the original time and amplitude, respectively, *i* is the amount of original data and *m* is the end. The specific process is as follows:

First, the beginning and end of initialization are presented as Equation (12):

(12) $$\left\{\begin{array}{l} E(1,1)=T_{1};E(1,2)=P_{1}\\ E(d,1)=T_{m};E(d,2)=P_{m} \end{array}\right.$$

where E(.) is the extremum matrix and *d* is the number of extremum points. Then, judging from the given conditions: when $P_{i-1}>P_{i}>P_{i+1}$ is satisfied, it is the minimum point; when $P_{i-1}<P_{i}<P_{i+1}$ is satisfied, it is the maximum point. The corresponding extremum point $T_{i}$ and $P_{i}$ are stored in E(.).

The above two steps are the basic principle and implementation process of extremum extraction.

**Figure 7:**
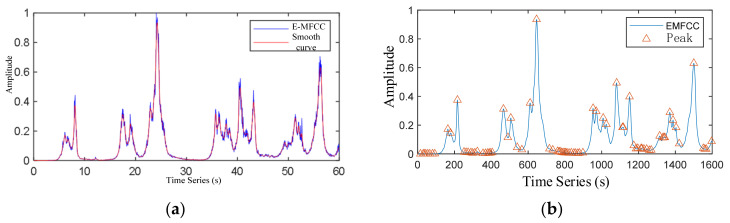
E-MFCC characteristic curve and extremum extraction. (**a**) E-MFCC characteristic curve and its smoothed characteristic curve and (**b**) E-MFCC extremum extraction.

The extracted E-MFCC features and the smoothed E-MFCC features are shown in Figure 7, where the peak represents the extreme, and the waveform after extremum extraction of smoothed E-MFCC characteristic curve is shown in Figure 7b.

#### 2.2.3. Formation and Combination of Triangular Waves

The formation and combination of triangular waves mainly includes four parts: triangular wave formation, combination on triangular wave, combination under triangular wave and expansion of frame width.

**Figure 8:**
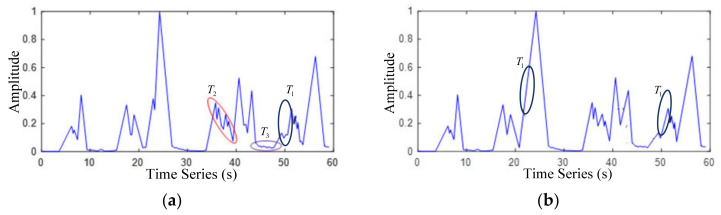

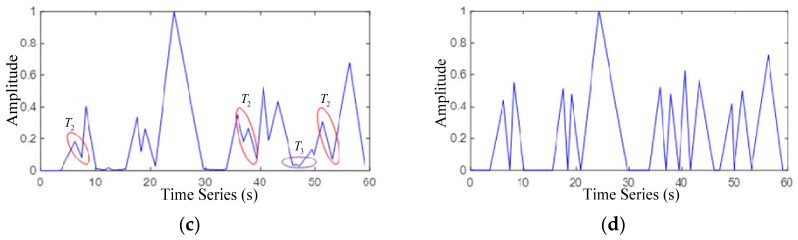
Formation and combination of triangular waves. (**a**) triangular wave formation, (**b**) combination on triangular wave, (**c**) combination under triangular wave and (**d**) frame width extension.

*T*_1_ is the interference triangle wave which appears in monotonic increasing, *T*_2_ is the interference triangle wave which appears in monotonic decreasing and *T*_3_ is the interference triangle wave which includes *T*_1_ and *T*_2_. In order to achieve satisfactory detection accuracy, it is necessary to carry out triangle wave up combinations, triangle wave down combinations and frame width expansion for these three cases. Figure 8b represents the combination of triangular waves, which is the first step of the combination rule of triangular waves. It is a solution to the *T*_1_ situation. Comparing Figure 8b with Figure 8a, the monotone increasing interference triangular wave of type *T*_1_ has been eliminated after the combination operation on the triangular wave. Figure 8c is the second step of the combination rule of triangular wave. It is a solution to *T*_2_ case. After the combination of triangular wave up and down processing, the peak value of some triangular waves is still very small; that is, the signal in some environmental noise segments is very weak. Comparing Figure 8c with Figure 8d, it can be figured out that after the operation of triangle waves lowers, the combination algorithm, the monotone decreasing interference triangle wave of type *T*_2_ has been eliminated. At the same time, after the combination of up and down triangle waves, *T*_3_ interference wave is basically eliminated. The waveform formed by frame width expansion is shown in Figure 8d. The frame width expansion algorithm is designed to solve the problem of abnormal noise (whistle, birdsong). The core idea is to expand the frame width of overlapped congested segment. By setting the minimum frame length of the congested segment, the traffic volume is obtained. The triangle wave analysis algorithm solves the problem of separating overlapping vehicle sections and can be applied. 

### 2.3. Experiment and Empirical Cases

#### 2.3.1. Experimental Data Acquisition

The Nanbin Road segment between the Caiyuanba Yangtze River Bridge and Chongqing Yangtze River Bridge was selected as the experimental data acquisition section. The entire road lays out from west to east along the Yangtze River (the north side adjacent to the river), with a total length around 1.3 km, and the south side is surrounded by mountains with no redundant branch section. The segment is intentionally selected to avoid pedestrian noise disturbance, as no shops, restaurants etc. are nearby, thus ensuring the quality of traffic noise collection. For urban arterial roads, noise intervention for pedestrians and noise intervention for shops are included and the data is collected from factors such as congested roads, etc. The data collection is seriously disturbed, so the two-lane off-peak hours are selected to collect the traffic noise. The driving noise signal can be converted from the time domain to the frequency domain by the fast Fourier transform (FFT), and then the spectrogram in the signal is extracted and analyzed. 

**Figure 9:**
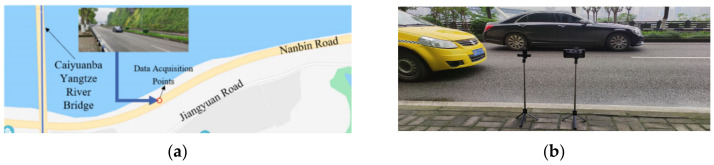
Schematic diagram of data acquisition. (**a**) selection of data acquisition points and (**b**) data acquisition (left bracket: a recording pen, right bracket: a smartphone).

The data collection points and collection schematic diagram are shown in Figure 9. As shown in Figure 9b, the equipment used in the study included a recording pen, a mobile phone and a computer. The recording pen is used for lossless recording with a 1536 KBPS/48 kHz sampling rate, and the recording format is WAV, supporting line-in recording and built-in microphone recording. The sensitivity of the microphone is −58 ± 3 dB, the working temperature is −25~70 °C and it has full directivity, electret type capacitor microphone-to-text. The frame length is set to 25 ms (namely 1200 sample points) and the frame shift is set to 12.5 ms. The video data were synchronously collected by the smart phone to compare the actual traffic volume. A video corresponding to the same segment will be recorded using the smartphone, to obtain the statistics of the vehicles on the road, which will be verified as the actual traffic volume of the experiment. Then, save the recorded traffic noise data in WAV format, and save the video data in MP4 format as a reference to obtain accurate traffic volume. The collected data were processed by Premiere Pro CC 2017 to remove the reverse traffic noise data and count the actual traffic volume for 60 min. The final synthetic sample was named ‘traffic noise’, and the actual traffic volume is 667 pcu.

#### 2.3.2. Evaluating Indicator

**Figure 10:**
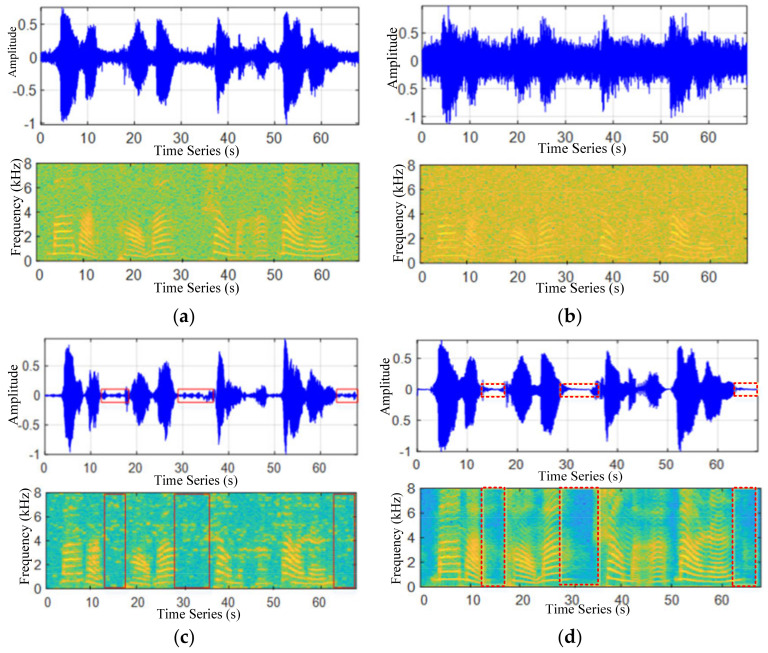
Noise reduction algorithm sonogram and waveform comparison. (**a**) original driving noise signal and its spectrogram, (**b**) noised-driving noise signal and its spectrogram, (**c**) spectral subtraction driving noise signal and its spectrogram and (**d**) improved spectral subtraction for driving noise signal and its spectrogram.

According to Ma et al. [31], the traffic detection evaluation indexes in this study were selected as traffic detection accuracy $r_{c}$, false detection rate $r_{w}$ and missed detection rate $r_{m}$, with definitions provided in Equation (13) below:

(13) $$\left\{\begin{array}{l} r_{c}=\frac{n_{\left(t,c\right)}}{n_{a}}\times 100\%\\ r_{w}=\frac{n_{t}-n_{\left(t,c\right)}}{n_{a}}\times 100\%\\ r_{m}=\frac{n_{a}-n_{t}-n_{\left(t,w\right)}}{n_{a}}\times 100\% \end{array}\right.$$

where *n_a_* is the actual number of vehicles in video synchronous acquisition data, *n_t_* is the total number of vehicles detected by traffic noise, *n*_(*t, c*)_ is the correct number of vehicles in the total number of detected vehicles, *n*_(*t*, *w*)_ is the number of vehicles wrongly detected from the noise signal, *n*_(*t*, *w*)_ = *n_t_* − *n*_(*t*, *c*)_ and *n_m_* is the number of undetected vehicles. The basic spectral subtraction and improved spectral subtraction are used to filter and denoise a section of original traffic noise signal, as shown in Figure 10.

Background from the environmental noise (distant vehicle) is superimposed. For Figure 10c, it can be seen that the traffic noise after noise reduction by spectral subtraction has an obvious discrimination compared with the original waveform, in which noise is marked in the red rectangular box. For Figure 10d, after filtering and noise reduction, when using the improved spectral subtraction, the signal of the non-congested segment is close to zero within the time domain diagram, which is ignorable, as marked in the red dotted rectangular box. In the spectrogram, there is no frequency distribution in the non-vehicle section, and the spectrum of the vehicle noise signal section is smoother.

#### 2.3.3. Noise Reduction Performance Comparison

A traffic statistics system based on traffic noise signal is established by App Designer in MATLAB R2020a. Different frame lengths directly affect the accuracy of detection. With regard to TWA characteristics, the parameter setting of the minimum frame length of the traffic segment signal is the key to detect the traffic volume.

**Figure 11:**
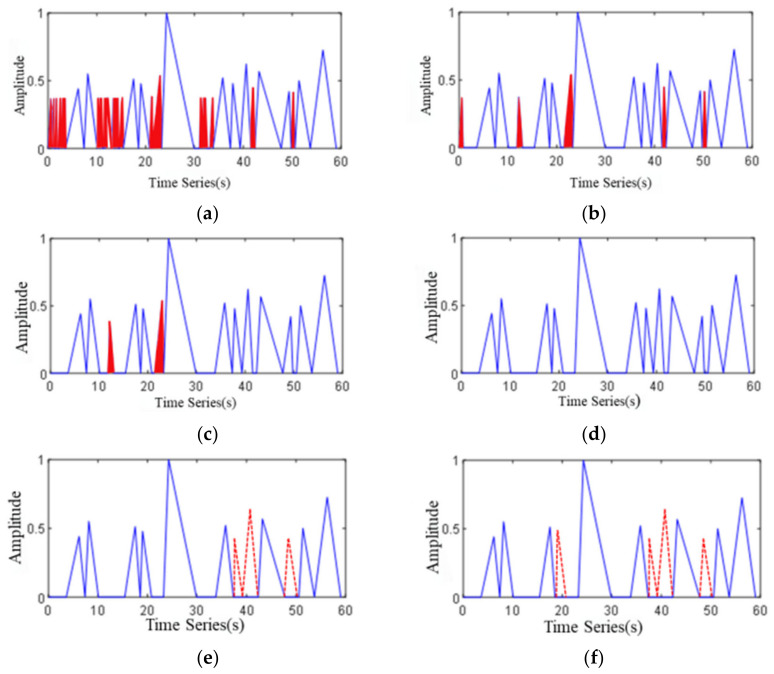
Comparison of the minimum frame length of different vehicle signal segments. (**a**) 10-frame, (**b**) 20-frame, (**c**) 30-frame, (**d**) 40-frame, (**e**) 50-frame and (**f**) 60-frame.

Taking 10 frames as the initial minimum frame length of the vehicle signal segment, 20 frames are successively increased to verify the traffic noise data of a segment with a traffic flow of 12 vehicles, as presented in Figure 11. In the Figure, the red triangle represents the triangular waves detected more than once, whereas the triangular box with a red dotted line represents the triangular waves missed. As can be figured out from Figure 11a–c, with the increase of frame length, the number of detected triangular waves becomes closer to the real number of vehicles, and the number of detected triangular waves decreases from six to two. In Figure 11d, when attaining 40 frames, the number of detected triangular waves equals the real vehicles. As the transition of frames increases, few triangular waves are detected and three vehicles missed detection at 50 frames and four vehicles missed detection at 60 frames. That is to say, when the minimum frame length of the vehicle signal section is set within certain range, the number of vehicles can be accurately detected. Through multiple experiments and comparisons, the minimum frame length of the vehicle signal segment was set within the range of 35–45 frames.

#### 2.3.4. Establishment of Simulation Platform

First, the platform selection area for traffic noise signal acquisition and feature extraction operation plate, acoustic signal acquisition acoustic signal reading are determined. Then, noise signal denoising and preprocessing occurs, followed by the selection of a traffic detection algorithm, which is divided into the STE detection algorithm, MFCC detection algorithm and TWA algorithm.

**Figure 12:**
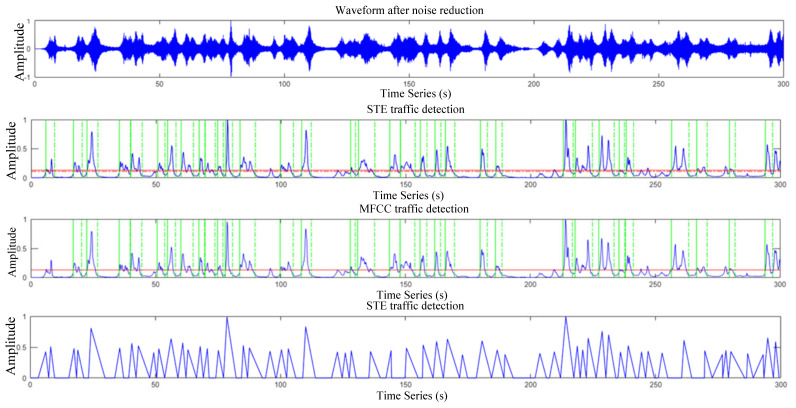
Waveform after noise reduction and three traffic detection characteristic curves.

Among the three traffic detection algorithms, traffic detection based on STE needs to consider the selection of high and low thresholds. Traffic detected by the three detection algorithms is presented in Figure 12. Referring to the threshold parameter selection method of endpoint detection in Zhang and Pan [32], the high and low threshold parameters were set as 0.13 and 0.11, respectively, in order to obtain the number of vehicles closer to the field situations. TWA is used for traffic detection, and the minimum length of vehicle signal segment is set as 40 frames, and other parameters are the same as MFCC detection. 

#### 2.3.5. Test Results and Analysis

**Figure 13:**
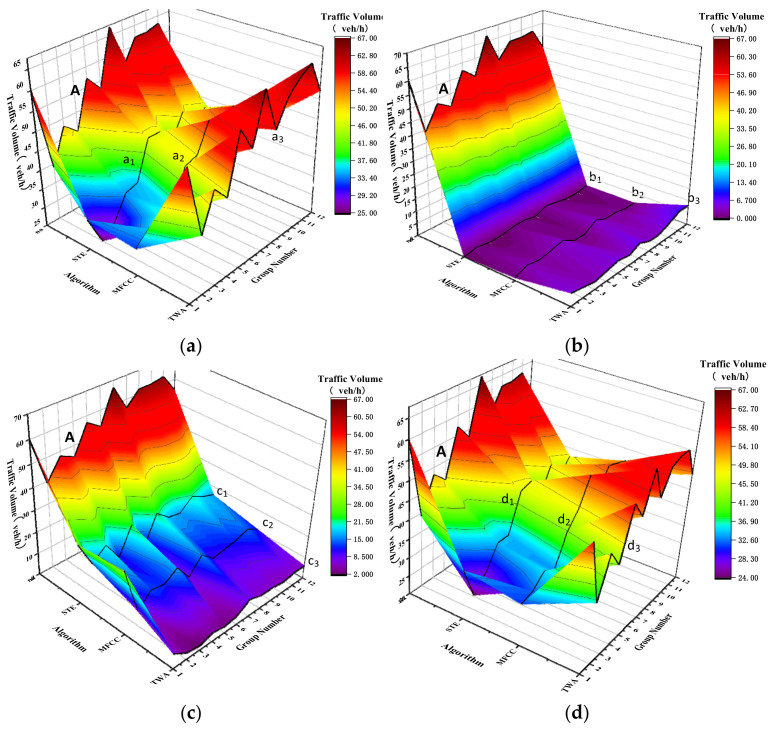
Analysis diagram of detection results of different algorithms. (**a**) *n_t_* comparison, (**b**) *n*_(*t,w*)_ comparison, (**c**) *n_m_* comparison and (**d**) *n*_(*t,c*)_ comparison.

Since the collected traffic noise data are large, the 60 min traffic noise signal acquisition data are divided into 1 to 12 groups with the length of 300 s in each group. Figure 13 represents the detection results of the first group of noise signals, with the statistical results provided in Table 1.

Table 1 presents the statistical results of 12 groups of different detection algorithms, in which $n_{\left(•\right)}^{t}$,$n_{\left(•\right)}^{m}$ and $n_{\left(•\right)}^{t}$ represent the corresponding numr of vehicles of STE, MFCC and TWA algorithms, respectively. The corresponding 3D views are provided in Figure 13.

The broken line A within the Figure is the distribution broken line of *n_a_*, a_1_, a_2_ and a_3_ represent the *n_t_* distribution under the action of STE, MFCC and TWA methods; b_1_, b_2_ and b_3_ represent the *n_(t,w)_* distribution under the action of STE, MFCC and TWA methods; c_1_, c_2_ and c_3_ represent the *n_m_* distribution under the action of STE, MFCC and TWA methods; d_1_, d_2_ and d_3_ represent the *n_(t,c)_* distribution under the action of STE, MFCC and TWA methods. From the Figure 13, the distributions of *n_t_* and *n*_(*t,c*)_ were found to be the closest to that of the broken with the lowest *n_m_* distribution. The statistical results of the three detection algorithms are shown in Table 2.

In the second column of Table 2, the vehicle missing rate of the three methods are provided. The average missing rate of TWA is 9%; that of MFCC is 23.69%; and that of STE is 32.23%, $R_{1}^{T}<R_{1}^{M}<R_{1}^{S}$.

In the third column of the table, the vehicle error detection rate of the three methods is provided, in which the average error detection rate of TWA is 9.15%; that of MFCC is 3.75%; and that of STE is 1.05%, $R_{2}^{S}<R_{2}^{M}<R_{2}^{T}$. In the fourth column of the table, the vehicle accuracy of the three methods was provided, in which the average accuracy of TWA attains 91%, much higher than that of MFCC (76.01%) and STE (67.77%), $R_{3}^{S}<R_{3}^{M}<R_{3}^{T}$. TWA can better achieve the detection of overlapping car bodies and has good stability within the time-varying environment.

## 3. Conclusions and Future Research Directions

This paper proposes a novel traffic noise analysis method for on-road traffic detection. The innovative feature parameter E-MFCC was defined for the fusion of STE and MFCC principal component features. Then, the extremum point was identified by exploring the entire characteristic curve, in which the overlapped segment signals were separated by triangular wave algorithms. The final triangular wave number is the number of vehicles detected in the running noise signal, which has theoretical and practical significance to expand and improve current traffic detection technologies, e.g., by loop detector or video camera sensors.

The average accuracy of STE, MFCC and TWA were calculated as 67.77%, 76.01% and 91%, respectively, indicating that TWA has rather good accuracy and is effective to detect traffic volume. Although the results are promising, this study indeed has limitations with regards to the data and the approach. First, this study only focuses on one-way lane traffic detection, without considering the two-way lane, which may lead to underutilization of information. In the follow-up work, the applicability of TWA in multi-lane processes will be investigated. Furthermore, during traffic noise pretreatment and feature extraction, window function, frame length, frame shift and feature dimension were mostly selected by personal experiences, without a set of mature theories or methods to guide the parameters’ configuration. Different parameter settings may help to shed lights on investigating the result of the study. Driving sound is not the end of traffic detection, because different models need to be converted into Passenger Car Units to get more accurate traffic statistics; therefore, the next part of the study will involve vehicle type recognition research based on the driving voice, and theoretical research efforts are required to improve result quality in the field. 

## Figures and Tables

**Table 1:** Results of three traffic detection algorithms.

Group	$n_{a}$	$n_{t}^{s}$	$n_{\left(t,w\right)}^{s}$	$n_{m}^{s}$	$n_{t}^{m}$	$n_{\left(t,w\right)}^{m}$	$n_{m}^{m}$	$n_{t}^{t}$	$n_{\left(t,w\right)}^{t}$	$n_{m}^{t}$
1	60	33	0	27	32	1	29	58	5	7
2	39	25	1	15	35	3	7	40	4	3
3	48	28	0	20	37	2	13	49	3	2
4	45	34	0	11	36	3	12	45	4	4
5	57	35	1	23	45	4	16	59	5	3
6	53	45	1	9	46	1	8	53	4	4
7	67	45	0	22	53	1	15	65	6	8
8	56	41	2	17	50	4	10	54	4	6
9	62	47	1	16	53	2	11	60	4	6
10	62	41	0	21	52	2	12	63	6	5
11	63	45	0	18	50	1	14	65	8	6
12	55	40	1	16	45	1	11	57	8	6

**Table 2:** Comparison of detection results of different algorithms.

Methods	Missed Detection Rate	False Detection Rate	Accuracy
STE	32.23%	1.05%	67.77%
MFCC	23.69%	3.75%	76.01%
TWA	9%	9.15%	91%

## Data Availability

Not applicable.
